# Natural products target the NRF2-ferroptosis axis in hepatocellular carcinoma: tumor sensitization versus hepatoprotection

**DOI:** 10.3389/fphar.2026.1968548

**Published:** 2026-10-22

**Authors:** Zugen Cheng, Ping Rong

**Affiliations:** 1 Kunming Children’s Hospital, Kunming, China; 2 Department of Pediatrics, General Hospital of Northern Theater Command, Shenyang, China

**Keywords:** drug resistance, ferroptosis, GPx4, hepatocellular carcinoma, KEAP1, natural products, Nrf2, SLC7A11

## Abstract

Hepatocellular carcinoma (HCC) remains a major cause of cancer mortality. First-line systemic therapy is now led by immune checkpoint inhibitor (ICI)-based regimens, notably atezolizumab plus bevacizumab and durvalumab plus tremelimumab, whereas sorafenib and lenvatinib are used mainly when immunotherapy is unsuitable and in later lines; resistance limits the durability of both strategies. Ferroptosis, an iron-dependent form of regulated cell death driven by phospholipid peroxidation, offers a potential strategy for overcoming this resistance. Nuclear factor erythroid 2-related factor 2 (NRF2) is a central but context-dependent regulator of this vulnerability. Transient NRF2 activation protects non-transformed hepatocytes from oxidative injury, whereas constitutive activation in HCC reinforces SLC7A11-and GPX4-dependent antioxidant defenses, promotes immune escape, and supports drug resistance. This mini-review evaluates how natural products and related formulations modulate this dual program. Polyphyllin I, tiliroside, ponicidin, picropodophyllin, curcumin, esculetin, sappanone A, and decursin act at distinct upstream nodes but converge on suppression of the p62-KEAP1-NRF2 pathway or its SLC7A11/GPX4 effectors, thereby restoring ferroptotic sensitivity in preclinical HCC models. In contrast, ginsenoside Rg1, thymoquinone, and hinokitiol activate NRF2 in models of non-malignant liver injury. Because the FSP1-CoQ10 and DHODH systems defend membranes against phospholipid peroxidation independently of GPX4, residual activity in these parallel arms can limit the efficacy of agents directed at the NRF2-SLC7A11-GPX4 axis. These findings support tumor-selective NRF2 modulation rather than indiscriminate systemic inhibition. Biased disruption of the KEAP1-p62 interaction and tumor-targeted nucleic-acid delivery provide proof of concept, but translation is constrained by limited pharmacokinetic, target-engagement, toxicologic, and clinical evidence.

## 1 Introduction

Hepatocellular carcinoma (HCC) is a leading cause of cancer-related mortality worldwide. Systemic treatment has since been reorganized around immunotherapy: current guidance places ICI-based combinations first in advanced disease, with atezolizumab plus bevacizumab and durvalumab plus tremelimumab established in randomized phase 3 trials and nivolumab plus ipilimumab added more recently (Finn et al., 2020; Abou-Alfa et al., 2022; Sangro et al., 2025; Yau et al., 2025). TKIs remain clinically important but are no longer first in sequence: sorafenib and lenvatinib are used when checkpoint inhibition is unsuitable, and lenvatinib, regorafenib, cabozantinib, and ramucirumab after progression on immunotherapy (Sangro et al., 2025). In both settings, benefit is frequently limited by primary or acquired resistance (Huang et al., 2021; Tu et al., 2024; Che et al., 2026). Ferroptosis is an iron-dependent form of regulated cell death in which unchecked phospholipid peroxidation disrupts membrane integrity. Because several TKIs intersect with redox and iron metabolism, restoring ferroptotic susceptibility has emerged as a plausible strategy for resistant HCC. Redox adaptation also bears on immunotherapy, because CD8^+^ T-cell-derived interferon gamma represses SLC7A11 and promotes tumor-cell ferroptosis (Wang W. et al., 2019), so the same defense program conditions responses to kinase inhibitors and to checkpoint blockade.

Nuclear factor erythroid 2-related factor 2 (NRF2) coordinates antioxidant and xenobiotic-stress responses. In non-transformed liver, transient NRF2 activation limits oxidative injury and fibrogenic signaling. In HCC, however, persistent NRF2 activity can support proliferation, immune evasion, and therapeutic resistance by reinforcing cystine uptake, glutathione synthesis, and phospholipid-hydroperoxide detoxification (Leung et al., 2020; Zhou et al., 2022; Gan et al., 2024). NRF2 is therefore a pharmacologically attractive but potentially hazardous target.

Natural products provide chemically diverse scaffolds and often engage multiple nodes within redox networks. Preclinical studies indicate that some natural products suppress NRF2 signaling to restore ferroptosis in HCC, whereas others activate NRF2 to protect injured hepatocytes (Fu et al., 2021; Yang C. et al., 2023; Zhou H. et al., 2024). This mini review examines that contextual duality, evaluates the strength and limitations of the pharmacological evidence, and considers how tumor-selective modulation might convert NRF2 from a conceptual paradox into a tractable therapeutic strategy. Figure 1 contrasts the two programs and places them within the wider network of ferroptosis defenses.

**FIGURE 1:**
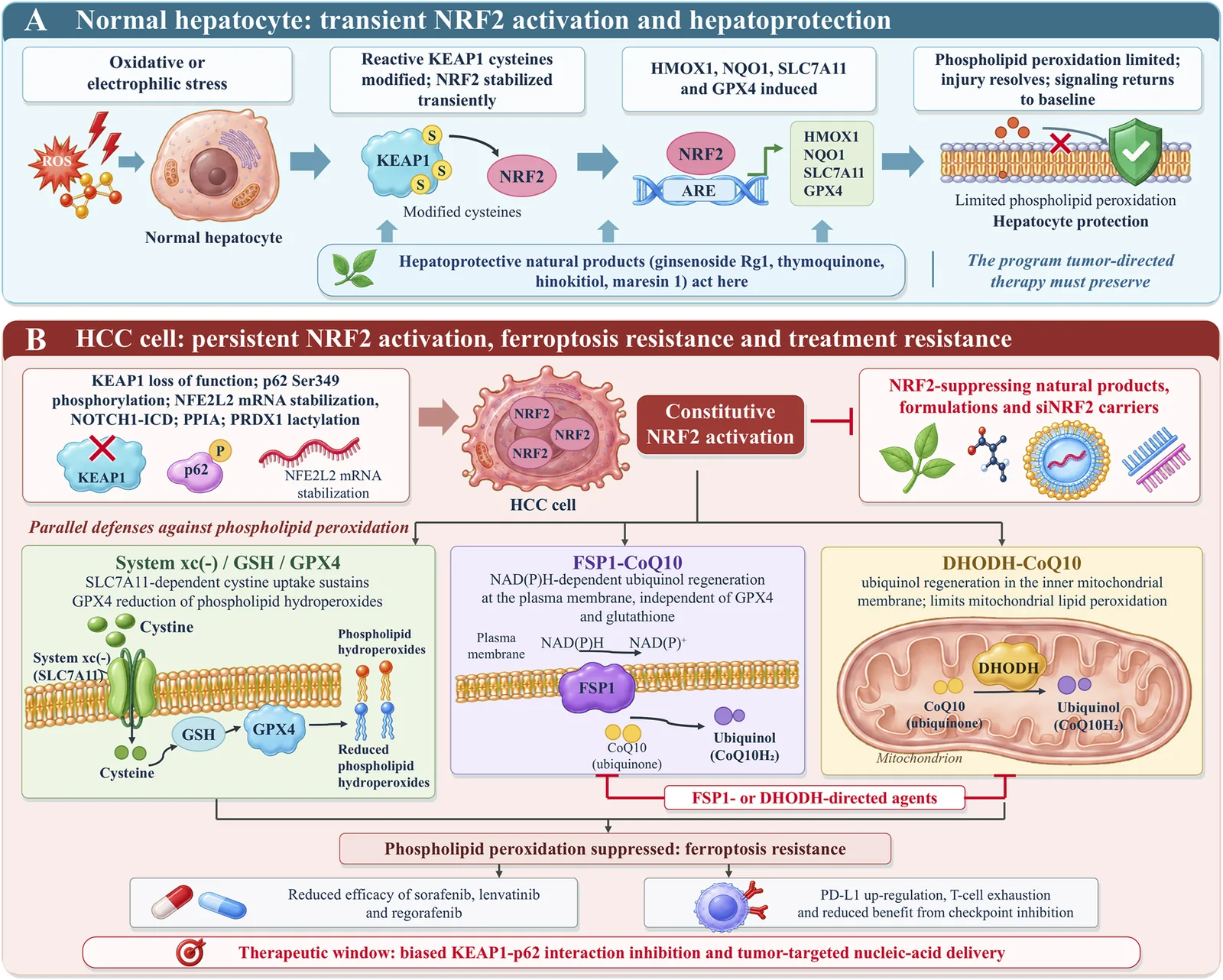
Context-dependent NRF2 activity and the network of ferroptosis defenses in the liver. **(A)** In non-transformed hepatocytes, oxidative or electrophilic stress modifies reactive KEAP1 cysteines and stabilizes NRF2 transiently, inducing cytoprotective genes (HMOX1, NQO1, SLC7A11, GPX4) so that phospholipid peroxidation is limited, injury resolves, and signaling returns to baseline; hepatoprotective natural products act in this compartment, which defines the program that tumor-directed therapy must preserve. **(B)** In HCC, KEAP1 loss of function, p62 Ser349 phosphorylation, and NFE2L2 mRNA stabilization produce constitutive NRF2 activation, which reinforces three partly independent defenses against phospholipid peroxidation: the SLC7A11-glutathione-GPX4 axis, the FSP1-CoQ10 system at the plasma membrane, and DHODH-dependent ubiquinol regeneration in the inner mitochondrial membrane. Ferroptosis resistance follows, together with reduced efficacy of kinase inhibitors and an immune phenotype characterized by PD-L1 upregulation and T-cell exhaustion. Residual FSP1 or DHODH activity can sustain protection when the NRF2-SLC7A11-GPX4 axis alone is disrupted, which is why agents directed at these arms are shown separately. Representative compounds, formulations, and delivery systems are listed in Table 1. CoQ10, coenzyme Q10; DHODH, dihydroorotate dehydrogenase; FSP1, ferroptosis suppressor protein 1; GSH, glutathione; HCC, hepatocellular carcinoma; NAD(P)H, reduced nicotinamide adenine dinucleotide (phosphate); xc (-), system xc (-).

Several recent syntheses cover adjacent ground. Wang Y. et al. (2026) recently surveyed NRF2 signaling in HCC with an emphasis on dual roles, epigenetic reprogramming, and metabolic vulnerability, and other recent reviews have addressed ferroptosis mechanisms in HCC (Tu et al., 2024), ferroptotic mechanisms of sorafenib resistance (Che et al., 2026), post-translational control of ferroptosis defense (Chen J. K. et al., 2026), and the dual role of NRF2 in HCC development and immune evasion (Gan et al., 2024). The present article shares the premise that NRF2 is context-dependent and that ferroptosis resistance is metabolically reinforced, but its unit of analysis is the pharmacological agent rather than the pathway: it appraises compound-level evidence for natural products, multi-herb formulations, and natural-product-derived delivery systems, including target-engagement quality, chemical standardization, and exposure-response data. It also treats tumor sensitization and hepatoprotection as a single therapeutic-window problem, examining compounds that activate NRF2 in injured liver alongside those that suppress it in tumors, rather than as an off-target concern. Readers seeking a comprehensive account of NRF2 transcriptional and epigenetic biology in HCC should consult Wang Y. et al. (2026); the present contribution is complementary and pharmacological.

## 2 Review approach

The source review used a focused narrative search of PubMed, Web of Science Core Collection, and Embase for peer-reviewed literature published from January 2015 through January 2026. PubMed and publisher records were additionally searched through 14 August 2026 to capture recent studies. The same sources were re-screened through 21 September 2026 during revision, adding the clinical-guidance, GPX4-independent-defense, and tumor-immune literature cited below; work appearing after that date is not covered. Search concepts combined (NRF2 OR NFE2L2 OR KEAP1) with (ferroptosis OR lipid peroxidation OR GPX4 OR SLC7A11 OR FSP1 OR AIFM2 OR DHODH), (hepatocellular carcinoma OR liver cancer OR liver injury), and (natural product OR herbal OR phytochemical OR traditional medicine OR nanoparticle). Primary studies were prioritized when they linked a defined compound, formulation, delivery system, or endogenous mediator to NRF2-dependent control of ferroptosis in HCC cells or hepatocytes. Reviews were used for context, and reference lists were screened for pivotal earlier studies. Records without mechanistic evidence involving the NRF2 pathway were not emphasized. Because the aim was a concise, critical synthesis rather than exhaustive evidence aggregation, the review was not registered and did not include PRISMA-based selection or formal risk-of-bias assessment.

## 3 NRF2 in the liver: adaptive cytoprotection and oncogenic dependence

### 3.1 KEAP1-NRF2 signaling and hepatoprotection

Under basal conditions, Kelch-like ECH-associated protein 1 (KEAP1) recruits NRF2 to a Cullin 3 ubiquitin ligase complex, promoting proteasomal degradation. Oxidative or electrophilic stress modifies reactive KEAP1 cysteines, allowing NRF2 to accumulate, enter the nucleus, and induce cytoprotective genes, including HMOX1/HO-1, NQO1, SLC7A11, and GPX4 (Zhou et al., 2022; Gan et al., 2024). This response is protective when it is transient and appropriately scaled (Figure 1A). Maresin 1 reduced ferroptotic liver injury through NRF2/HO-1/GPX4 signaling (Yang W. et al., 2022); ginsenoside Rg1 promoted KEAP1 degradation and NRF2 nuclear translocation in a chronic lipopolysaccharide injury model (Zhou H. et al., 2024); and Gardeniae Fructus extract attenuated thioacetamide-induced fibrosis through AMPK/SIRT1 and NRF2-related signaling (Shin et al., 2021). These models establish a physiological rationale for NRF2 activation outside malignant tissue.

### 3.2 Constitutive activation, ferroptosis resistance, and immune escape

In HCC, sustained NRF2 activation shifts this adaptive response toward oncogenic dependence (Figure 1B). NRF2 increases SLC7A11-mediated cystine uptake and supports GPX4 activity, thereby preserving glutathione-dependent clearance of phospholipid hydroperoxides (Wang Q. et al., 2021; Qu et al., 2025; Xing et al., 2024). NRF2 also regulates drug transport and metabolic programs, including ABCC5-associated resistance (Huang et al., 2021). HO-1 is context-dependent because heme catabolism can enhance antioxidant adaptation yet also expand the labile iron pool; its direction of effect should therefore be established experimentally rather than inferred from expression alone (Xiu et al., 2022; Yang R. et al., 2023).

Multiple upstream lesions converge on NRF2. KEAP1 loss of function provides a direct route, whereas phosphorylated p62/SQSTM1 competitively occupies KEAP1 and prevents NRF2 turnover under oxidative or drug pressure (Ren et al., 2021; Tang et al., 2022). Mitochondrial TSPO can promote p62 accumulation by disrupting autophagic flux (Zhang D. et al., 2023); IGF2BP3 stabilizes m6A-modified NFE2L2 mRNA (Lu et al., 2022); the NOTCH1 intracellular domain interferes with the KEAP1-NRF2 interaction (Chang et al., 2025); and DDX5 deficiency increases NRF2 expression through Wnt/beta-catenin and non-canonical NF-kappaB signaling (Li Z. et al., 2024). Aristolochic acids provide a carcinogenic example by altering p53-dependent transcription and suppressing ferroptosis (Hou et al., 2025).

NRF2 also intersects with antitumor immunity. TSPO-driven NRF2 activity increased PD-L1 expression in HCC models (Zhang D. et al., 2023), while PP1A-dependent KEAP1 dephosphorylation was linked to NRF2 activation, PD-L1 expression, T-cell exhaustion, and reduced efficacy of lenvatinib plus immune-checkpoint blockade (Zhou J. et al., 2025).

### 3.3 Defenses that operate in parallel to GPX4

Because NRF2-directed strategies are usually read out through SLC7A11 and GPX4, defenses that operate without glutathione peroxidase activity require explicit treatment. Ferroptosis suppressor protein 1 (FSP1, encoded by AIFM2) is myristoylated at the plasma membrane, where it reduces ubiquinone to ubiquinol, regenerating a lipophilic radical-trapping antioxidant independently of glutathione and GPX4 (Bersuker et al., 2019; Doll et al., 2019). Dihydroorotate dehydrogenase (DHODH) performs an analogous reduction of coenzyme Q in the inner mitochondrial membrane and restrains mitochondrial lipid peroxidation, particularly when GPX4 is inhibited (Mao et al., 2021). Both systems are coupled to the pathway considered here: in KEAP1-inactive tumors, constitutive NRF2 activity raises FSP1 expression and creates a targetable CoQ-FSP1 dependency (Koppula et al., 2022), so suppressing NRF2 may lower this arm without eliminating it. In liver cancer specifically, lysyl oxidase-like 3 stabilizes DHODH and restrains mitochondrial ferroptosis to promote chemoresistance (Zhan et al., 2023). Residual FSP1 or DHODH activity is therefore a parsimonious explanation for incomplete ferroptosis after disruption of the NRF2-SLC7A11-GPX4 axis, and studies of the compounds below should report these effectors, or ubiquinol-dependent rescue, rather than GPX4 alone (Figure 1B).

## 4 Natural products engaging the NRF2-ferroptosis axis

The available preclinical literature can be organized into two pharmacological programs: NRF2 suppression to sensitize malignant cells to ferroptosis and NRF2 activation to protect non-transformed liver. Table 1 summarizes representative compounds, formulations, delivery systems, and mechanistic comparators.

**TABLE 1:** Representative natural products, formulations, delivery systems, and mechanistic comparators that modulate the NRF2-ferroptosis axis in liver models.

Compound or preparation	Source or class	NRF2 effect	Reported mechanism and outcome	References
*NRF2 suppression: ferroptosis sensitization in HCC*				
Polyphyllin I	Paris polyphylla Sm. (Melanthiaceae)	Down	Suppresses NRF2/HO-1/GPX4; increases iron accumulation, GSH depletion, and mitochondrial injury	Yang R. et al., 2023
Tiliroside	Flavonoid glycoside; widely distributed	Down	Inhibits TBK1; reduces p62 Ser349 phosphorylation; restores KEAP1-dependent NRF2 turnover; sensitizes to sorafenib	Yang C. et al., 2023
Ponicidin	Isodon rubescens (Hemsl.) H.Hara (Lamiaceae)	Down	Stabilizes the KEAP1-NRF2 complex and blocks nuclear translocation in lenvatinib-resistant cells	Zhang L. et al., 2025
Picropodophyllin	Cyclolignan; natural IGF1R inhibitor	Down	Inhibits PI3K/AKT; promotes NRF2 degradation; reduces SLC7A11 and SLC40A1	Zheng et al., 2025
Curcumin	Curcuma longa L. (Zingiberaceae)	Down	Reduces p62 and NRF2 while increasing KEAP1; promotes ferroptosis	Deng et al., 2026
Decursin	Angelica gigas Nakai (Apiaceae)	Down	Attenuates NRF2/GPX4; active in xenografts and sorafenib-resistant cells; SLC11A2 link remains computational	Li J. et al., 2026
Esculetin	Naturally occurring coumarin; present in Fraxinus spp.	Down	Inhibits NRF2-xCT/GPX4; increases Fe2+, ROS, and lipid peroxidation	Qu et al., 2025
Sappanone A	Biancaea sappan (L.) Tod. (Fabaceae)	Down	Suppresses NRF2/xCT/GPX4; increases Fe2+ and lipid peroxides	Xing et al., 2024
Withaferin A	Withania somnifera (L.) Dunal (Solanaceae)	Down	Links NRF2-regulated EMT, ferroptosis, metastatic behavior, and drug resistance	Zhang Y. et al., 2022
Caryophyllene oxide	Sesquiterpene oxide; widely distributed	Indirect	Induces NCOA4/FTH1/LC3-dependent ferritinophagy and expands the labile iron pool	Xiu et al., 2022
Metformin + sorafenib	Synthetic comparator	Down	Engages p62-KEAP1-NRF2/HO-1 and enhances ferroptosis	Tang et al., 2022
Hua Zheng San Ji Fang	Multi-herb formula; composition as reported	Down	Increases KEAP1 and suppresses NRF2/HO-1	Su et al., 2025
Benja-ummarit	Thai polyherbal formula	Down	Reduces CD98, NRF2, and GPX4; increases KEAP1; induces iron- and oxidation-dependent death	Sandech et al., 2024
Salvia nanovesicles	Salvia chinensis Benth. (Lamiaceae)	Down	Engages AMPK/NRF2/xCT; promotes ferroptosis	Liang et al., 2025
Scutellaria nanovesicles	Scutellaria barbata D. Don (Lamiaceae)	Down	Engages NRF2/SLC7A11/GPX4; DIA proteomics	Liang et al., 2026
Pt (IV)-18beta-glycyrrhetinic-acid platform	Hybrid natural-product/prodrug nanoplatform	Down	Combines NRF2 suppression with apoptosis and ferroptosis	Chen X. et al., 2026
siNRF2 + sorafenib nanocarrier	Synthetic comparator	Down	NRF2 knockdown with TKI; marked tumor inhibition *in vivo*	Tong et al., 2023
p53 mRNA + NRF2 siRNA LNP	Targeted synthetic comparator	Down	Lactobionic-acid targeting; restores ferroptosis and apoptosis in resistant HCC	Liao et al., 2026
*GPX4-independent defenses: FSP1 and DHODH*				
Ginsenoside (20)S-APPT	Panax ginseng C.A.Mey. (Araliaceae); protopanaxatriol-type derivative	FSP1-directed	Targets FSP1 to induce ferroptosis in HCC and cholangiocarcinoma cells	Liu F.-Y. et al., 2025
Oridonin derivative (G6PD/PGD modulator)	Isodon rubescens (Hemsl.) H.Hara (Lamiaceae); semisynthetic	FSP1/DHODH-directed	Modulates G6PD/PGD to overcome FSP1/DHODH-mediated defense; suppresses liver cancer	Ma et al., 2026
Sorafenib + FSP1 inhibitor co-delivery	Synthetic comparator	FSP1-directed	Dual ferroptosis in tumor cells and immunosuppressive macrophages; enhances immunotherapy	Tang C. et al., 2025
*NRF2 activation: hepatoprotection in non-transformed liver*				
Ginsenoside Rg1	Panax ginseng C.A.Mey. (Araliaceae)	Up	Promotes KEAP1 degradation and NRF2 nuclear translocation; inhibits inflammasome activation	Zhou H. et al., 2024
Gardeniae Fructus extract	Gardenia jasminoides J.Ellis (Rubiaceae)	Up	Engages AMPK/SIRT1 and NRF2-related signaling; attenuates thioacetamide-induced fibrosis	Shin et al., 2021
Thymoquinone	Nigella sativa L. (Ranunculaceae)	Up	Inhibits KEAP1 and promotes NRF2 expression and nuclear translocation across injury models	Talebi et al., 2020
Hinokitiol	Tropolone derivative found in Cupressaceae species	Up	Activates NRF2/GPX4; reduces hepatocyte ferroptosis in MASH	Yin et al., 2025
Maresin 1	Endogenous DHA-derived lipid mediator	Up	Activates NRF2/HO-1/GPX4 and reduces ROS in ferroptotic liver injury	Yang W. et al., 2022
*References case: carcinogen-driven NRF2 activation*				
Aristolochic acids	Aristolochia spp. (Aristolochiaceae)	Up (adverse)	Alter p53-dependent transcription, increase NRF2, inhibit ferroptosis, and promote tumor growth	Hou et al., 2025

Direction refers to reported NRF2 signaling. Evidence is predominantly preclinical and should not be interpreted as clinical efficacy. Botanical nomenclature follows the source reports and commonly accepted current usage. DIA, data-independent acquisition; DHA, docosahexaenoic acid; DHODH, dihydroorotate dehydrogenase; EMT, epithelial-mesenchymal transition; FSP1, ferroptosis suppressor protein 1; GSH, glutathione; HCC, hepatocellular carcinoma; LNP, lipid nanoparticle; MASH, metabolic dysfunction-associated steatohepatitis; ROS, reactive oxygen species; TKI, tyrosine kinase inhibitor.

### 4.1 Suppressing the p62-KEAP1-NRF2 pathway

Chemically unrelated compounds converge on NRF2 through distinct upstream targets. Polyphyllin I reduced NRF2/HO-1/GPX4 signaling and produced iron accumulation, glutathione depletion, and mitochondrial injury in HCC models (Yang R. et al., 2023). Tiliroside inhibited TBK1, reduced p62 Ser349 phosphorylation, weakened the p62-KEAP1 interaction, and sensitized HCC to sorafenib (Yang C. et al., 2023). Ponicidin was reported to stabilize the KEAP1-NRF2 complex and inhibit NRF2 nuclear translocation in lenvatinib-resistant cells (Zhang L. et al., 2025). Picropodophyllin inhibited IGF1R/PI3K/AKT signaling, promoted NRF2 degradation, and reduced SLC7A11 and SLC40A1 expression (Zheng et al., 2025). Curcumin likewise shifted the p62-KEAP1-NRF2 pathway toward ferroptosis (Deng et al., 2026).

Recent evidence adds decursin to this group. Multi-omics analysis and xenograft experiments showed attenuation of the NRF2-GPX4 program, inhibition of tumor growth, and retained activity in sorafenib-resistant HCC cells (Li J. et al., 2026). Computational analyses implicated SLC11A2 in iron handling, but this proposed target requires direct validation. Together, these studies support the pathway as a shared pharmacological node while illustrating a recurring limitation: pathway modulation is often better established than direct molecular binding.

### 4.2 Convergence on SLC7A11, GPX4, and iron handling

The system Xc-/glutathione/GPX4 axis converts diverse upstream signals into a common ferroptosis threshold. Esculetin inhibited NRF2-xCT/GPX4 signaling and increased Fe2+, reactive oxygen species, and lipid peroxidation (Qu et al., 2025), whereas sappanone A produced a similar response through NRF2/xCT/GPX4 (Xing et al., 2024). Caryophyllene oxide approached the same endpoint from the iron side by inducing NCOA4/FTH1/LC3-dependent ferritinophagy and expanding the labile iron pool (Xiu et al., 2022). Withaferin A further linked NRF2-regulated epithelial-mesenchymal transition with ferroptosis and drug resistance (Zhang Y. et al., 2022).

Natural-product pharmacology has also begun to address these arms directly. Ginsenoside (20)S-APPT induced ferroptosis in HCC and cholangiocarcinoma cells by targeting FSP1 (Liu et al., 2025); a semisynthetic oridonin derivative overcame FSP1- and DHODH-mediated defense by modulating G6PD and PGD in liver cancer models (Ma et al., 2026); and co-delivery of sorafenib with an FSP1 inhibitor induced ferroptosis in tumor cells and in immunosuppressive macrophages, improving responses to immunotherapy in mice (Tang C. et al., 2025). These agents are complementary to the NRF2-directed compounds above, and combinations covering both the glutathione-dependent and the ubiquinol-dependent arms are a logical next step.

Evidence quality is not uniform. Increased reactive oxygen species or reduced viability alone does not establish ferroptosis; stronger studies combine lipid-peroxidation measurements with iron dependence, characteristic ultrastructural or biochemical changes, and rescue by a ferroptosis inhibitor (Tu et al., 2024). This distinction is important when comparing natural products whose pleiotropic effects may activate apoptosis, autophagy, or ferroptosis simultaneously.

### 4.3 Multi-component formulations and targeted delivery

Multi-component formulations broaden potential pathway coverage but introduce challenges in chemical standardization, constituent attribution, pharmacokinetics, and interaction analysis. Hua Zheng San Ji Fang increased KEAP1 and suppressed NRF2/HO-1 signaling (Su et al., 2025), while the Thai formulation Benja-ummarit reduced CD98, NRF2, and GPX4 and induced an iron- and oxidation-dependent death phenotype (Sandech et al., 2024). These findings are hypothesis-generating until active constituents, exposure-response relationships, and reproducible quality controls are defined.

Delivery systems may provide a more direct solution to the selectivity problem. Exosome-like nanovesicles from Salvia chinensis and Scutellaria barbata promoted ferroptosis through AMPK/NRF2/xCT or NRF2/SLC7A11/GPX4 signaling, respectively (Liang et al., 2025; 2026). A self-assembled Pt (IV)-18beta-glycyrrhetinic-acid platform combined NRF2 inhibition with apoptosis and ferroptosis (Chen X. et al., 2026). Beyond natural-product carriers, co-delivery of siNRF2 and sorafenib inhibited tumor growth *in vivo* (Tong et al., 2023), and lactobionic-acid-functionalized lipid nanoparticles co-delivering p53 mRNA and NRF2 siRNA restored ferroptosis in resistant HCC (Liao et al., 2026). These systems remain preclinical, but they demonstrate how spatial targeting can separate antitumor NRF2 inhibition from systemic redox toxicity.

### 4.4 Activating NRF2 for hepatoprotection

The opposite intervention is rational in non-malignant liver injury. Ginsenoside Rg1 promoted KEAP1 degradation and NRF2 nuclear translocation while suppressing NLRP3, NLRP1, and AIM2 inflammasome activation (Zhou H. et al., 2024). Thymoquinone has been reported to inhibit KEAP1 and promote NRF2 expression and nuclear translocation across organ-injury models (Talebi et al., 2020), and hinokitiol activated NRF2/GPX4 to reduce hepatocyte ferroptosis in metabolic dysfunction-associated steatohepatitis (Yin et al., 2025). Maresin 1 provides non-botanical corroboration of the same protective program (Yang W. et al., 2022). These findings do not justify unselected systemic NRF2 activation in patients with established HCC; instead, they define the normal-tissue function that tumor-directed inhibition must preserve (Figure 1A).

## 5 Discussion

### 5.1 Reframing the double-edged-sword problem

The apparent contradiction between NRF2 activation and inhibition is resolved by context. Transient activation in injured hepatocytes restores redox homeostasis, whereas persistent activation in malignant cells creates a survival dependency (Leung et al., 2020; Zhou et al., 2022; Gan et al., 2024). Cell identity, activation duration, disease stage, and the surrounding immune and metabolic environment are therefore more informative than the direction of NRF2 change alone. Viral hepatitis adds further complexity because hepatitis B and C viruses can use NRF2-associated redox adaptation to support replication and pro-survival signaling (Kalantari et al., 2023). The relevant therapeutic question is not whether NRF2 is beneficial or harmful, but where, when, and in which molecularly defined tumors it should be modulated.

### 5.2 NRF2 as a convergence node in kinase inhibitor and immunotherapy resistance

Several resistance programs converge on NRF2 or its effectors. In sorafenib-resistant HCC, ABCC5 stabilizes SLC7A11 (Huang et al., 2021), SLC27A5 loss maintains glutathione through NRF2/GSR (Xu et al., 2023), and GSTZ1 loss reduces ferroptotic sensitivity through NRF2/GPX4 (Wang Q. et al., 2021). In lenvatinib resistance, SERPINE2 activates JAK2/STAT3, promotes NRF2 nuclear translocation, and increases GCLC transcription (Liu et al., 2026), while PP1A acts through KEAP1 dephosphorylation (Zhou J. et al., 2025). ZNF207-driven PRDX1 lactylation further links metabolic adaptation to NRF2 activation and regorafenib resistance (Yang T. et al., 2025). This convergence supports NRF2 as a candidate bottleneck, but predictive biomarkers and prospective clinical validation are lacking.

The same node bears on immunotherapy, which now leads first-line care. NRF2 activity driven by mitochondrial TSPO increased PD-L1 expression (Zhang D. et al., 2023), and PP1A-dependent KEAP1 dephosphorylation was associated with NRF2 activation, T-cell exhaustion, and reduced efficacy of lenvatinib plus checkpoint blockade (Zhou J. et al., 2025). Conversely, activated CD8^+^ T cells repress SLC7A11 and sensitize tumor cells to ferroptosis (Wang W. et al., 2019), and dual ferroptosis induction in tumor cells and immunosuppressive macrophages improved immunotherapy in preclinical HCC (Tang C. et al., 2025). NRF2-directed agents should therefore be tested in immunocompetent models with immune endpoints, both as potential sensitizers of checkpoint inhibition and as agents whose activity may depend on an intact T-cell response.

### 5.3 Post-translational control of the NRF2 network

Post-translational regulation is an important but incompletely mapped layer. Phosphorylation, ubiquitination, acetylation, and lactylation can alter NRF2 stability or modify upstream proteins that control its localization and transcriptional output (Chen J. K. et al., 2026). PRDX1 lactylation at Lys67 enhances NRF2 nuclear translocation (Yang T. et al., 2025), whereas cathepsin S promotes KEAP1 degradation through the autophagy-lysosome system and thereby reduces NRF2 turnover (Xu et al., 2025). These findings should not be conflated with direct modification of NRF2 itself. Defining the relevant enzymes, temporal sequence, and reversibility of these events could expose targets that are more selective than global NRF2 blockade.

### 5.4 Defining a therapeutic window

Two preclinical strategies illustrate how selectivity might be achieved. A biased KEAP1-p62 protein-protein interaction inhibitor preferentially disrupted KEAP1 binding to phosphorylated p62 in p62-aberrant HCC while largely preserving basal KEAP1-NRF2 regulation (Zhao et al., 2026). Alternatively, tumor-targeted nanocarriers can localize NRF2-directed nucleic acids and combination therapy (Tong et al., 2023; Liao et al., 2026). The first strategy exploits a tumor-specific pathway configuration; the second exploits spatial delivery. Neither yet establishes clinical feasibility, but together they offer a rational framework for widening the therapeutic window.

### 5.5 Translational priorities and conclusion

Translation requires a more pharmacological evidence base. Most natural-product studies remain cell-based, and relatively few integrate exposure-response relationships, bioavailability, metabolism, normal-liver toxicity, and direct target-engagement assays. Molecular docking or network pharmacology should generate hypotheses rather than substitute for biophysical binding, genetic epistasis, or target-rescue experiments. Multi-herb formulations additionally require chemical fingerprinting, batch consistency, and constituent-level interaction analysis.

Four priorities follow. First, NRF2-pathway biomarkers, including KEAP1 alteration, p62 abundance, and modification patterns, should be tested for patient stratification. Second, tumor and adjacent liver should be evaluated together to quantify the therapeutic window. Third, ferroptosis readouts should routinely include the GPX4-independent arms described in Section 3.3, so that residual FSP1 or DHODH activity and ACSL4-LPCAT3-dependent substrate supply are measured rather than inferred (Che et al., 2026; Chen J. K. et al., 2026). Fourth, combination studies should include immune endpoints because NRF2 can influence PD-L1 expression and checkpoint-therapy response (Zhang D. et al., 2023; Zhou J. et al., 2025).

In conclusion, natural products are useful pharmacological probes and potential lead compounds, not yet validated NRF2-directed therapies for HCC. Their strongest collective signal is mechanistic convergence: diverse compounds repeatedly reduce the p62-KEAP1-NRF2/SLC7A11/GPX4 defense program and restore ferroptotic susceptibility in preclinical models. Progress now depends on converting this convergence into tumor-selective, exposure-defined, and biomarker-guided interventions while preserving the hepatoprotective NRF2 response in non-malignant tissue. This pharmacological framing is the contribution intended here, and it separates the present synthesis from recent pathway-centered reviews of NRF2 and of ferroptosis in HCC (Gan et al., 2024; Tu et al., 2024; Che et al., 2026; Chen J. K. et al., 2026; Wang Y. et al., 2026): the question addressed is not only how NRF2 governs ferroptosis, but which agents, at which exposures and with what selectivity, can lower the ferroptotic threshold in tumor tissue while preserving the same program in non-malignant liver. Whether that separation can be achieved in patients, and whether it survives the GPX4-independent defenses described in Section 3.3, is the central open question.

## References

[B1] Abou-Alfa G. K. Lau G. Kudo M. Chan S. L. Kelley R. K. Furuse J. (2022). Tremelimumab plus durvalumab in unresectable hepatocellular carcinoma. NEJM Evid. 1, EVIDoa2100070. 10.1056/EVIDoa2100070 38319892

[B2] Bersuker K. Hendricks J. M. Li Z. Magtanong L. Ford B. Tang P. H. (2019). The CoQ oxidoreductase FSP1 acts parallel to GPX4 to inhibit ferroptosis. Nature 575, 688–692. 10.1038/s41586-019-1705-2 31634900 PMC6883167

[B3] Chang C. Wang M. Li J. Qi S. Yu X. Xu J. (2025). Targeting NOTCH1-KEAP1 axis retards chronic liver injury and liver cancer progression via regulating stabilization of NRF2. J. Exp. Clin. Cancer Res. 44, 232. 10.1186/s13046-025-03488-3 PMC12335071 40781716

[B4] Che L. Zhu L. Zhou L. Zhou Y. (2026). Deciphering sorafenib resistance in hepatocellular carcinoma via ferroptotic mechanisms. Biochim. Biophys. Acta Rev. Cancer 1881, 189566. 10.1016/j.bbcan.2026.189566 41763496

[B5] Chen J. K. Li L. Fang S. Zhou H. (2026). Post-translational modification networks in ferroptosis: orchestrating defense, drug resistance, and therapeutic opportunities in hepatocellular carcinoma. J. Hepatocell. Carcinoma 13, 33. 10.2147/JHC.S607656 PMC13200707 42199338

[B6] Chen X. Cui S. Deng R. Zhang H. Zhang Z. Zhang Y. (2026). A highly biocompatible self-assembled Pt(IV) nanoplatform integrating Nrf2 inhibition for synergistic apoptosis and ferroptosis in hepatocellular carcinoma. Mat. Today Bio 38, 103138. 10.1016/j.mtbio.2026.103138 PMC13134034 42079500

[B7] Deng J. Wu Z. Ning S. Xu C. Liu J. Yu Y. (2026). Curcumin induces ferroptosis in hepatocellular carcinoma by regulating the P62-KEAP1-NRF2-signaling pathway. BMC Cancer 26, 90. 10.1186/s12885-025-15307-1 PMC12822296 41316098

[B8] Doll S. Freitas F. P. Shah R. Aldrovandi M. da Silva M. C. Ingold I. (2019). FSP1 is a glutathione-independent ferroptosis suppressor. Nature 575, 693–698. 10.1038/s41586-019-1707-0 31634899

[B9] Finn R. S. Qin S. Ikeda M. Galle P. R. Ducreux M. Kim T.-Y. (2020). Atezolizumab plus bevacizumab in unresectable hepatocellular carcinoma. N. Engl. J. Med. 382, 1894–1905. 10.1056/NEJMoa1915745 32402160

[B10] Fu K. Wang C. Ma C. Zhou H. Li Y. (2021). The potential application of Chinese medicine in liver diseases: a new opportunity. Front. Pharmacol. 12, 771459. 10.3389/fphar.2021.771459 PMC8600187 34803712

[B11] Gan L. Wang W. Jiang J. Tian K. Liu W. Cao Z. (2024). Dual role of Nrf2 signaling in hepatocellular carcinoma: promoting development, immune evasion, and therapeutic challenges. Front. Immunol. 15, 1429836. 10.3389/fimmu.2024.1429836 PMC11402828 39286246

[B12] Hou C.-Y. Suo Y.-H. Lv P. Yuan H.-F. Zhao L.-N. Wang Y.-F. (2025). Aristolochic acids-hijacked p53 promotes liver cancer cell growth by inhibiting ferroptosis. Acta Pharmacol. Sin. 46, 208–221. 10.1038/s41401-024-01354-0 PMC11696298 39090392

[B13] Huang W. Chen K. Lu Y. Zhang D. Cheng Y. Li L. (2021). ABCC5 facilitates the acquired resistance of sorafenib through the inhibition of SLC7A11-induced ferroptosis in hepatocellular carcinoma. Neoplasia 23, 1227–1239. 10.1016/j.neo.2021.11.002 PMC8591347 34768109

[B14] Kalantari L. Rostami Ghotbabadi Z. Gholipour A. Ehymayed H. M. Najafiyan B. Amirlou P. (2023). A state-of-the-art review on the NRF2 in hepatitis virus-associated liver cancer. Cell Commun. Signal. 21, 318. 10.1186/s12964-023-01351-6 PMC10633941 37946175

[B15] Koppula P. Lei G. Zhang Y. Yan Y. Mao C. Kondiparthi L. (2022). A targetable CoQ-FSP1 axis drives ferroptosis- and radiation-resistance in KEAP1 inactive lung cancers. Nat. Commun. 13, 2206. 10.1038/s41467-022-29905-1 PMC9033817 35459868

[B16] Leung H.-W. Lau E. Y. Leung C. O. N. Lei M. M. L. Mok E. H. K. Ma V. (2020). NRF2/SHH signaling cascade promotes tumor-initiating cell lineage and drug resistance in hepatocellular carcinoma. Cancer Lett. 476, 48–56. 10.1016/j.canlet.2020.02.008 32061952

[B17] Li Z. Kim W. Utturkar S. M. Yan B. Lanman N. A. Elzey B. D. (2024). DDX5 deficiency drives non-canonical NF-kappaB activation and NRF2 expression, influencing sorafenib response and hepatocellular carcinoma progression. Cell Death Dis. 15, 583. 10.1038/s41419-024-06977-z PMC11315975 39122708

[B18] Li J. Wang Z. Wei K. Liu Q. Liang L. Lin J. (2026). Decursin induces ferroptosis via the NRF2/GPX4/SLC11A2 axis and suppresses migration in hepatocellular carcinoma. Biomed. Pharmacother. 195, 118941. 10.1016/j.biopha.2025.118941 41496356

[B19] Liang Z. Fang X. Zhang Z. Tao M. Gong W.-H. Jian H. (2025). Natural exosome-like nanovesicles from Salvia chinensis Benth induce ferroptosis to suppress hepatocellular carcinoma progression via AMPK/Nrf2/xCT axis. Cell. Signal. 134, 111928. 10.1016/j.cellsig.2025.111928 40484104

[B20] Liang Z. Fang X. Tao M. Zhang Z. Tan X. Zeng P. (2026). DIA-based proteomic analysis reveals Scutellaria barbata D. Don exosome-like nanovesicles promote ferroptosis in HCC through Nrf2/SLC7A11/GPX4 pathway. Funct. Integr. Genomics 26, 111. 10.1007/s10142-026-01892-2 42209785

[B21] Liao Y. Zeng X. Zhang X. Hu Y. Zhao Y. Luo Q. (2026). Optimized lipid nanoparticles for co-delivery of mRNA and siRNA therapeutics in refractory liver cancer. Adv. Mat. 38, e19473. 10.1002/adma.202519473 41510588

[B22] Liu F.-Y. Yang Y.-J. Wang X.-L. Hong Y. Ou-Yang X.-P. Chang L. (2025). Ginsenoside (20)S-APPT induces ferroptosis in hepatocellular carcinoma and cholangiocarcinoma by targeting FSP1. Acta Pharmacol. Sin. 46, 3273–3290. 10.1038/s41401-025-01589-5 PMC12644808 40550962

[B23] Liu K. Huang S. Xu Y. Mao Y. Chen N. Yuan Y. (2026). SERPINE2-mediated activation of JAK2/STAT3 facilitates NRF2 nuclear translocation and GCLC transcription to confer ferroptosis resistance and lenvatinib resistance in hepatocellular carcinoma. J. Exp. Clin. Cancer Res. 45, 174. 10.1186/s13046-026-03746-y PMC13459319 42251352

[B24] Lu Z. Yang H. Shao Y. Sun W. Jiang Y. Li J. (2022). IGF2BP3-NRF2 axis regulates ferroptosis in hepatocellular carcinoma. Biochem. Biophys. Res. Commun. 627, 103–110. 10.1016/j.bbrc.2022.08.040 36030651

[B25] Ma C. Han L. Yao H. Zhao W. Chen F. Wu X. (2026). Modulating G6PD/PGD to overcome FSP1/DHODH-mediated ferroptosis defence: a novel oridonin derivative suppresses liver cancer. Br. J. Pharmacol. 183, 249–267. 10.1111/bph.70160 40859915

[B26] Mao C. Liu X. Zhang Y. Lei G. Yan Y. Lee H. (2021). DHODH-mediated ferroptosis defence is a targetable vulnerability in cancer. Nature 593, 586–590. 10.1038/s41586-021-03539-7 PMC8895686 33981038

[B27] Qu Z. Zeng J. Zeng L. Li X. Zhang F. (2025). Esculetin triggers ferroptosis via inhibition of the Nrf2-xCT/GPx4 axis in hepatocellular carcinoma. Chin. J. Nat. Med. 23, 443–456. 10.1016/S1875-5364(25)60853-3 40274347

[B28] Ren X. Li Y. Zhou Y. Hu W. Yang C. Jing Q. (2021). Overcoming the compensatory elevation of NRF2 renders hepatocellular carcinoma cells more vulnerable to disulfiram/copper-induced ferroptosis. Redox Biol. 46, 102122. 10.1016/j.redox.2021.102122 PMC8416961 34482117

[B29] Sandech N. Yang M. C. Juntranggoor P. Rukthong P. Gorelkin P. V. Savin N. (2024). Benja-ummarit induces ferroptosis with cell ballooning feature through ROS and iron-dependent pathway in hepatocellular carcinoma. J. Ethnopharmacol. 335, 118672. 10.1016/j.jep.2024.118672 39127118

[B30] Sangro B. Argemi J. Ronot M. Paradis V. Meyer T. Mazzaferro V. (2025). EASL clinical practice guidelines on the management of hepatocellular carcinoma. J. Hepatol. 82, 315–374. 10.1016/j.jhep.2024.08.028 39690085

[B31] Shin M.-R. Lee J. A. Kim M. J. Lee S. H. Oh M. Moon J. (2021). Gardeniae fructus attenuates thioacetamide-induced liver fibrosis in mice via both AMPK/SIRT1/NF-kappaB pathway and Nrf2 signaling. Antioxidants 10, 1837. 10.3390/antiox10111837 PMC8614944 34829709

[B32] Su B. Wang S. Xiong Z. Liu T. Leng Y. Deng H. (2025). Hua zheng san Ji Fang promotes ferroptosis through Keap1/Nrf2 pathway in hepatocellular carcinoma cells. J. Ethnopharmacol. 352, 120165. 10.1016/j.jep.2025.120165 40543691

[B33] Talebi M. Talebi M. Farkhondeh T. Samarghandian S. (2020). Biological and therapeutic activities of thymoquinone: focus on the Nrf2 signaling pathway. Phytother. Res. 35, 1739–1753. 10.1002/ptr.6905 33051921

[B34] Tang K. Chen Q. Liu Y. Wang L. Lu W. (2022). Combination of metformin and sorafenib induces ferroptosis of hepatocellular carcinoma through p62-Keap1-Nrf2 pathway. J. Cancer 13, 3234–3243. 10.7150/jca.76618 PMC9475364 36118519

[B35] Tang C. He C. Wang D. Guo J. Yin X. Ye H. (2025). Co-delivery of sorafenib and an FSP1 inhibitor triggers dual ferroptosis in tumor cells and immunosuppressive macrophages for enhanced immunotherapy in mouse models of hepatocellular carcinoma. Nat. Commun. 16, 10096. 10.1038/s41467-025-65056-9 PMC12627561 41253833

[B36] Tong R. Feng X. Sun J. Ling Z. Wang J. Li S. (2023). Co-delivery of siNRF2 and sorafenib by a “click” dual functioned hyperbranched nanocarrier for synergistically inducing ferroptosis in hepatocellular carcinoma. Small 20, e2307273. 10.1002/smll.202307273 38102096

[B37] Tu S. Zou Y. Yang M. Zhou X. Xu Z. Jiang Y. (2024). Ferroptosis in hepatocellular carcinoma: mechanisms and therapeutic implications. Biomed. Pharmacother. 182, 117769. 10.1016/j.biopha.2024.117769 39689515

[B38] Wang W. Green M. Choi J. E. Gijón M. Kennedy P. D. Johnson J. K. (2019). CD8+ T cells regulate tumour ferroptosis during cancer immunotherapy. Nature 569, 270–274. 10.1038/s41586-019-1170-y 31043744 PMC6533917

[B39] Wang Q. Cheng B. Xue Q. Gao Q. Huang A. Wang K. (2021). GSTZ1 sensitizes hepatocellular carcinoma cells to sorafenib-induced ferroptosis via inhibition of NRF2/GPX4 axis. Cell Death Dis. 12, 426. 10.1038/s41419-021-03718-4 PMC8087704 33931597

[B40] Wang Y. Gao L. Yang H. Ouyang D. Zhu J. Jian W. (2026). The NRF2 signaling pathway in hepatocellular carcinoma: dual roles, epigenetic reprogramming, and therapeutic opportunities in metabolic vulnerability. Front. Oncol. 16, 1874956. 10.3389/fonc.2026.1874956 PMC13487741 42621384

[B41] Xing Y. Yang H. Dai C.-L. Qiu Z. Guan Y.-Y. Zhang L. (2024). Investigating the mechanism of ferroptosis induction by sappanone A in hepatocellular carcinoma: NRF2/xCT/GPX4 axis. Eur. J. Pharmacol. 983, 176965. 10.1016/j.ejphar.2024.176965 39214275

[B42] Xiu Z. Zhu Y. Han J. Li Y. Li Y. Yang X. (2022). Caryophyllene oxide induces ferritinophagy by regulating the NCOA4/FTH1/LC3 pathway in hepatocellular carcinoma. Front. Pharmacol. 13, 930958. 10.3389/fphar.2022.930958 PMC9313605 35899120

[B43] Xu F.-L. Wu X. Chen C. Wang K. Huang L. Xia J. (2023). SLC27A5 promotes sorafenib-induced ferroptosis in hepatocellular carcinoma by downregulating glutathione reductase. Cell Death Dis. 14, 22. 10.1038/s41419-023-05558-w PMC9837139 36635256

[B44] Xu R.-C. Sun J. Wang F. Liu H.-H. Qi Z. Shi X. (2025). Cathepsin S regulates ferroptosis sensitivity in hepatocellular carcinoma through the KEAP1-NRF2 signaling pathway. Redox Biol. 86, 103815. 10.1016/j.redox.2025.103815 PMC12357265 40784043

[B45] Yang C. Lu T. Liu M. Yuan X. Li D. Zhang J. (2023). Tiliroside targets TBK1 to induce ferroptosis and sensitize hepatocellular carcinoma to sorafenib. Phytomedicine 111, 154668. 10.1016/j.phymed.2023.154668 36657316

[B46] Yang W. Wang Y. Zhang C. Huang Y. Yu J. Shi L. (2022). Maresin1 protect against ferroptosis-induced liver injury through ROS inhibition and Nrf2/HO-1/GPX4 activation. Front. Pharmacol. 13, 865689. 10.3389/fphar.2022.865689 PMC9013935 35444546

[B47] Yang T. Zhang S. Nie K. Cheng C. Peng X. Huo J. (2025). ZNF207-driven PRDX1 lactylation and NRF2 activation in regorafenib resistance and ferroptosis evasion. Drug resist. updat. 82, 101274. 10.1016/j.drup.2025.101274 40680452

[B48] Yang R. Gao W. Wang Z. Jian H. Lian P. Yu X. (2023). Polyphyllin I induced ferroptosis to suppress the progression of hepatocellular carcinoma through activation of the mitochondrial dysfunction via Nrf2/HO-1/GPX4 axis. Phytomedicine 122, 155135. 10.1016/j.phymed.2023.155135 37856990

[B49] Yau T. Galle P. R. Decaens T. Sangro B. Qin S. da Fonseca L. G. (2025). Nivolumab plus ipilimumab versus lenvatinib or sorafenib as first-line treatment for unresectable hepatocellular carcinoma (CheckMate 9DW): an open-label, randomised, phase 3 trial. Lancet 405, 1851–1864. 10.1016/S0140-6736(25)00403-9 40349714

[B50] Yin X. Liu Z. Li C. Wang J. (2025). Hinokitiol ameliorates MASH in mice by therapeutic targeting of hepatic Nrf2 and inhibiting hepatocyte ferroptosis. Phytomedicine 139, 156472. 10.1016/j.phymed.2025.156472 39922149

[B51] Zhan M. Ding Y. Huang S. Liu Y. Xiao J. Yu H. (2023). Lysyl oxidase-like 3 restrains mitochondrial ferroptosis to promote liver cancer chemoresistance by stabilizing dihydroorotate dehydrogenase. Nat. Commun. 14, 3123. 10.1038/s41467-023-38753-6 PMC10229567 37253718

[B52] Zhang Y. Tan Y. Liu S. Yin H. Duan J. Fan L. (2022). Implications of withaferin A for the metastatic potential and drug resistance in hepatocellular carcinoma cells via Nrf2-mediated EMT and ferroptosis. Toxicol. Mech. Methods 33, 47–55. 10.1080/15376516.2022.2075297 35592903

[B53] Zhang D. Man D. Lu J. Jiang Y. Ding B. Su R. (2023). Mitochondrial TSPO promotes hepatocellular carcinoma progression through ferroptosis inhibition and immune evasion. Adv. Sci. 10, e2206669. 10.1002/advs.202206669 PMC10214260 36994647

[B54] Zhang L. Wang H. Liang B. Qin L. Zhang M. Lv X. (2025). Ponicidin promotes ferroptosis to enhance treatment sensitivity in lenvatinib-resistant hepatocellular carcinoma cells through regulation of KEAP1/NRF2. Phytomedicine 143, 156824. 10.1016/j.phymed.2025.156824 40382941

[B55] Zhao Z. Lu H. Zhang J. Y. Zhao J. Wang Y.-X. Zhu T. (2026). Discovery of a biased Kelch-like ECH-associated protein 1-p62 (Keap1-p62) protein-protein interaction inhibitor for the management of p62 aberrant hepatocellular carcinoma. J. Med. Chem. 69, 13141–13164. 10.1021/acs.jmedchem.6c00096 42216892

[B56] Zheng J. Liu Y. Zhu F. Liu S. Cai Z. Liu M. (2025). Picropodophyllin induces ferroptosis via blockage of AKT/NRF2/SLC7A11 and AKT/NRF2/SLC40A1 axes in hepatocellular carcinoma as a natural IGF1R inhibitor. Phytomedicine 143, 156840. 10.1016/j.phymed.2025.156840 40412057

[B57] Zhou J. Zheng Q. Chen Z. (2022). The Nrf2 pathway in liver diseases. Front. Cell Dev. Biol. 10, 826204. 10.3389/fcell.2022.826204 PMC8866876 35223849

[B58] Zhou H. Liu Y. Su Y. Ji P. Kong L. Sun R. (2024). Ginsenoside Rg1 attenuates lipopolysaccharide-induced chronic liver damage by activating Nrf2 signaling and inhibiting inflammasomes in hepatic cells. J. Ethnopharmacol. 324, 117794. 10.1016/j.jep.2024.117794 38244950

[B59] Zhou J. Gao M. Zhang S. Guo W.-W. He W. Zhang M. (2025). PP1A modulates the efficacy of lenvatinib plus ICIs therapy by inhibiting ferroptosis in hepatocellular carcinoma. Adv. Sci. 12, e2501730. 10.1002/advs.202501730 PMC12279197 40344394

